# Particulate matter removal by precipitation under normal and wildfire conditions across the United States using double machine learning

**DOI:** 10.21203/rs.3.rs-10855101/v1

**Published:** 2026-09-08

**Authors:** Dinesh Neupane, Viviana Maggioni, Lucas Henneman

**Affiliations:** 1 Department of Civil, Environmental and Infrastructure Engineering, George Mason University, Fairfax Virginia 22030; 2 Department of Civil and Environmental Engineering, Politecnico di Milano, Milano, Italy

**Keywords:** Wet deposition, particulate matter removal, wildfire smoke PM

## Abstract

Precipitation scavenging is an important sink for particulate matter (PM) and can therefore have direct consequences for human and ecosystem health. Controlled experiments and modeling studies quantifying precipitation-driven removal have been conducted in the past, but the literature lacks large-scale observational-based estimates of precipitation influence on PM concentrations. This gap has become more important as wildfire smoke now contributes a growing share of PM pollution across the United States, and climate change drives rainfall pattern shifts. We analyzed hourly PM_2.5_ and PM_10_ observations across the contiguous United States from 2014 to 2023 at 760 (PM_2.5_) and 451 (PM_10_) Environmental Protection Agency monitors. These records were merged with NLDAS-2 meteorology and NOAA HMS wildfire smoke classifications. We used a machine learning-based causal inference framework (DoubleML) to separate the effect of precipitation from confounding meteorological variables. Particle removal increased with rainfall intensity, particle size, and initial concentration, but declined as wildfire smoke loading increased. Removal rate became increasingly negative up to about 2-3 mm h^−1^ of rainfall, then declined at higher rainfall intensities even though total removal (i.e., total change in PM concentration from one hour to the next) increased in magnitude. We parameterized the results obtained from the DoubleML model into power law washout coefficients for PM_2.5_ (*a* = 0.0404, *b* = 0.5951) and for PM_10_ (*a* = 0.0812, *b* = 0.4508) to provide observation-based parameters that can be used in chemical transport models. The methodology and parameters can be adopted for human-PM exposure assessment, air quality model calibrations, and PM deposition to land, vegetation, and surface waters.

## Introduction

Many studies have shown that exposure to airborne particulate matter (PM) is connected to serious health risks, including increased risk of cardiovascular disease, premature mortality, cancer, and higher rates of adverse respiratory health indicators (Alemayehu, Asfaw, and Terfie 2020; Bell et al. 2007; U.S. Environmental Protection Agency 2022). Over the past few decades, the United States (U.S.) has made significant progress in reducing PM concentrations through regulatory measures and technological improvements (Clay and Muller 2019). However, the growing frequency and severity of wildfires are threatening to reverse these gains (Zhang et al. 2023b). Wildfire smoke contains high concentrations of fine PM, which can persist over large areas and elevate pollution levels for days or even weeks (Chen, Zhang, and Sheng 2025; Hung et al. 2021). These smoke events often lead to hazardous air quality, particularly in the western United States, presenting new challenges for both public health and air quality management. Wildfire smoke dominates summer PM_2.5_ concentrations in parts of the western U.S., with contributions exceeding 50% of seasonal mean concentrations during major fire years (O’Dell et al. 2019).

The removal of aerosol particles suspended in the atmosphere by precipitation is one of the major mechanisms for eliminating atmospheric aerosol particles (Chate et al. 2003). This process occurs via two main pathways: in-cloud scavenging, where particles are incorporated during cloud droplet formation, and below-cloud scavenging, where falling hydrometeors collect particles during descent. The efficiency of below-cloud scavenging depends on precipitation characteristics such as intensity, duration, and phase (e.g., rain, snow), as well as physicochemical properties of PM such as concentration, size distribution, and composition (Oduber et al. 2021; Petroff and Zhang 2010). As precipitation falls, droplets of diameter D_D_ interact with ambient PM, capturing particles through one of three mechanisms: impaction, where particles directly collide with the raindrop; interception, where particles follow airflow paths and adhere to the drop surface; and diffusion, where smaller PM particles randomly migrate and attach due to Brownian motion (Figure 1) (Seinfeld 2016; Slinn 1983).

During both typical conditions and wildfire events, particles can be scavenged by precipitation and deposited on land and surface waters which may alter PM concentrations in air along with water chemistry and ecosystem conditions. In urban environments, atmospheric deposition facilitates the transfer of polluting substances and materials in the atmosphere to the catchment surface through wet or dry deposition, with wet deposition contributing directly to the surface runoff pollution (Marsalek 2014). Deposition can be a source of stormwater pollutants including total suspended solids (TSS), nutrients (N and P) and metals such as Cu, Pb and Zn (Campbell et al. 2026; Clay and Muller 2019). Aerosol particles removed by rainfall can accumulate on impervious surfaces and flow through stormwater runoff into nearby streams and rivers (EPA 2015).

Many lab and field studies on precipitation and air quality use scavenging coefficients (Λ, s^−1^), a first-order parameter that represents the fractional rate at which particles are washed out by precipitation. The composition of an aerosol particle influences how it interacts with water. For example, hygroscopic particles are removed as compared to hydrophobic particles which tend to persist longer until aging makes them easier to remove (Bae, Jung, and Pyo Kim 2006; Sakata and Asakura 2007; Wang, Zhang, and Moran 2010). However, calculating scavenger coefficients typically requires knowledge of collision efficiency and raindrop size distribution, which are not available widely and cannot be directly applied to air quality models (Guo et al. 2016). Rather than relying on scavenging coefficients, it is more practical to connect air quality to measurable meteorological factors like rainfall characteristics (QuÈrel et al. 2014). The knowledge gap is even larger under wildfire conditions. Smoke from wildfires produces much higher PM concentrations and introduces a different particle composition compared to typical urban or industrial pollution. In a study by Sparks and Wagner (2021), particles enriched in Cu, Zn, Sn, and Pb were observed in wildfire samples, but not in non-wildfire samples. Changes in composition and particle size distribution change the solubility of particles, which changes their propensity to remain in raindrops. Despite the growing importance of wildfire smoke as a pollution source, evidence on how precipitation interacts with smoke is limited.

The primary objective of this study is to improve estimates of PM scavenging values for different rainfall intensities in both typical conditions and periods affected by wildfire smoke. We used hourly PM_2.5_ and PM_10_ (i.e., particulate matter with an aerodynamic diameter between 2.5 and 10 *μm*) concentrations from 760 and 451 locations, respectively, paired with several meteorological factors and wildfire smoke categories across the U.S. from 2014 to 2023. We aim to provide new insights into the propensity of rainfall to remove PM across precipitation intensities, baseline PM concentration, particle size, and wildfire smoke conditions. To achieve this, we used a double machine learning statistical approach to quantify the removal effects of rainfall on PM. Results from this large-scale observational study can inform preparations and interventions to mitigate the harmful effects on human health and ecosystem damage from wildfire smoke.

## Data and Methods

Figure 2 presents the workflow of this study which includes the acquisition of precipitation and meteorological data from the North American Land Data Assimilation System Phase 2 (NLDAS-2), NOAA Hazard Mapping System (HMS) Smoke Polygons, and Environmental Protection Agency Air Quality System (AQS). These inputs were used in the preliminary analysis and to model the aerosol removal effect of precipitation isolating the metrological drivers.

### Surface PM Observations

We quantified changes in particulate matter concentrations with PM_2.5_ and PM_10_ associated with precipitation across the contiguous U.S. from 2014 to 2023 using a 10-year hourly dataset. We used *in situ* hourly average PM observations from the EPA’s AQS monitoring network (U.S. Environmental Protection Agency n.d.; accessed October 15, 2024). The AQS network contains air pollution data collected by air quality monitors across the contiguous U.S. maintained by the EPA, state, local, and tribal agencies. Air quality monitoring networks including State or Local Air Monitoring Stations (SLAMS) and Special Purpose Monitors are used for non-regulatory purposes. For convenience, we refer to them as the “EPA monitors”. The EPA AQS database contains observations from both urban and rural monitoring sites, and the number of monitoring stations is different each year (Figure 3). In 2023, the datasets contained observations from 760 PM_2.5_ and 451 PM_10_ monitoring stations. Quality control procedures were applied to remove negative values and records that do not have valid PM measurements from AQS data prior to analysis.

### Metrological Data

NLDAS-2 is a full-coverage hourly gridded dataset derived from remotely sensed observations and reanalysis to support modeling and research applications. Historical NLDAS meteorological data were derived from the North American Regional Reanalysis (NARR) analysis fields, which have a 32 km spatial resolution and a 3-hourly temporal frequency (Mesinger et al. 2006). The NARR fields were spatially interpolated to the finer resolution of NLDAS (1/8-degree grid, about 12x12 km^2^) and then temporally disaggregated to the hourly frequency (Cosgrove et al. 2003). The gridded dataset is at 12x12 km^2^ spatial resolution and is reported daily with an approximately 4-day lag. These data are available from 1979 to the present.

We used available hourly meteorological variables from the NLDAS-2 that are relevant to PM and precipitation. The wet removal of aerosols is controlled by several meteorological factors, and the gridded NLDAS-2 dataset provides a spatially and temporally consistent set of variables that are not uniformly available from the EPA AQS meteorological records. These include temperature at 2 meters (TMP), surface pressure (PRES), specific humidity (SPFH), wind components at 10 meters (UGRD, VGRD), longwave and shortwave radiation (DLWRF, DSWRF), convective precipitation fraction (CONVfrac), total precipitation (APCP), potential evaporation (PEVAP), and convective available potential energy (CAPE). Wind speed (WV) was computed from the *u* and *v* components as $\sqrt[{2}]{UGRD^{2}+VGRD^{2}}$. These variables were matched to the monitoring locations using the containing NLDAS-2 grid cell. An overview of meteorological variables, units, and description is provided in supplemental information **Error! Reference source not found.**.

### Satellite Observation of Smoke Plumes

The NOAA HMS is an operational tool that relies on imagery from several satellites and trained satellite analysts to identify fire locations and smoke plumes across North America. HMS smoke plume polygons are derived from visible satellite imagery from the Geostationary Operational Environmental Satellite (GOES) satellites with an approximate spatial resolution of 4 to 10 km (Jerrett et al. 2005). The smoke plume polygons indicate locations where there is likely smoke somewhere in the atmospheric column during daylight hours rather than ground-level smoke concentrations. Smoke plumes are categorized into four levels based on visual density: No Smoke (NS), Light, Medium, and Heavy, with higher categories indicating greater smoke opacity and visibility reduction. Smoke plume information was assigned to the monitoring locations based on spatial-temporal overlap with the plume polygon. In cases where multiple plume densities were observed within a single hour, the highest intensity was used. In general, the number and extent of HMS smoke plumes are conservative estimates of smoke occurrence in the atmospheric column (Brey et al. 2018a).

### Local Spatial Clustering Analysis

We define the outcome variable *Y* for the primary analysis as removal efficiency, calculated as the percent reduction in PM concentration between consecutive hours t and t−1:

(1) $$Y=\left(\frac{{PM}_{t}-PM_{t-1}}{PM_{t-1}}\right)\times 100\%$$

A negative value means PM concentration decreased compared to the previous hour, whereas a positive value means it increased. Both conditions can occur in real scenarios. In an exploratory analysis, we quantified spatial clustering of PM removal efficiency associated with precipitation and other meteorological variables using local indicators of spatial association (LISA) (Anselin 1995). We calculated median site-specific removal efficiency for all hours with precipitation greater than 0.1 mm; the median represents the typical removal and limits the sensitivity to the extreme precipitation or smoke events. We used a K-nearest neighbor weighting scheme with eight neighbors per site to determine the spatial correlation among stations’ median removal efficiency. We used a common choice of k = 8 to balance spatial details and statistical stability (Getis and Ord 1992). The spatial weight matrix was standardized so that each observation has an equal contribution to local statistics.

We applied local Moran’s I to the median removal coefficients to detect local spatial autocorrelation. Local Moran’s I for station i was defined as:

(2) $$I_{i}=\left(y_{i}-\overset{‾}{y}\right)\sum_{j}w_{ij}\left(y_{j}-\overset{‾}{y}\right)$$

where $y_{i}$ is the station-median PM removal coefficient, $\overset{‾}{y}$ is the global mean, and $w_{ij}$ are row-standardized spatial weights with each row representing a focal station *i* and its neighboring stations j (Anselin 1995).

The statistical significance for this exploratory analysis was evaluated using a permutation-based p-value, and the locations were classified as significant at α=0.05. Monitors that met the significance level were assigned to one of four quadrants: two representing clusters of high and low PM removal efficiency, respectively, and two representing spatial outliers in which low values are surrounded by high values or vice versa.

### Causal Inference Using DoubleML

To estimate the effect of precipitation on PM by adjusting for non-linear relationships and confounding with meteorological variables, we implemented a Double Machine Learning (DoubleML) framework (Chernozhukov et al. 2018). DoubleML uses flexible machine learning models to isolate the effects of observed covariates from both the treatment and outcome. In this framework, hourly accumulated precipitation was the treatment variable *D*, PM removal efficiency was the outcome *Y*, and hourly meteorology and smoke presence were covariates *X*. Two nuisance functions g(X), which predict the outcome from the covariates, and m(X) which predicts the treatment from the covariates, were fitted as:

(3) g(X)=E[Y∣X],m(X)=E[D∣X]

We specified the outcome and treatment models as:

(4) D=m(X)+v,E[v∣X]=0

(5) Y=θ(X)D+g(X)+ε,E[ε∣X,D]=0

where error terms *ε* and *v* capture variation in the outcome and treatment variables that is not explained by the covariates and are assumed to be mean-independent of *X*. The parameter *θ*(*X*) represents the conditional average treatment effect (CATE) which is the expected change in PM removal efficiency due to precipitation under specific meteorological conditions(Medianovskyi et al. 2023).

The treatment variable *D* is hourly accumulated precipitation (APCP). The covariate vector *X* includes NLDAS-2 hourly meteorological variables Temperature (TEMP), Specific Humidity (SPFH), Surface Pressure (PRES), Zonal Wind Speed (U), Meridional Wind Speed (V), Downward Longwave Radiation (DLWRF), Downward Shortwave Radiation (DSWRF), Potential Evaporation (PEVAP), Convective Available Potential Energy (CAPE) and Wind Speed (WV). These covariates are the controls for confounding influences on both precipitation and PM concentrations. The parameter *θ*(*X*) represents the conditional average treatment effect and represents the expected change in removal efficiency due to precipitation under specified meteorological and smoke conditions.

Both g(X) and *m*(*X*) were estimated using Extreme Gradient Boosting (XGBoost) due to its ability to flexibly model nonlinear relationships and interactions. Hyperparameters were selected separately for the outcome and treatment models using inner K-fold cross-validation, where candidate XGBoost configurations were compared based on mean cross-validation RMSE. 5-fold cross validation was adopted in both models to reduce overfitting and ensure reliable estimates. The cross-validation step consisted of splitting the dataset into 5 non-overlapping subsets then, iteratively, using four subsets to train the model, then using the other to assess its performance (Anguita et al. 2012; T. -T. Wong and P. -Y. Yeh 2020). This process was repeated until each fold served once as the validation. The residual outcome and treatment terms were then constructed as:

(6) $$Y_{res}=Y-\overset{ˆ}{g}(X),\;D_{res}=D-\hat{m}(X)$$

Residuals represent the variation in PM removal and precipitation that remains after adjustment for the observed covariates. $Y_{\text{res}}$ isolated the portion of PM removal efficiency that cannot be explained by background meteorology, smoke density, and baseline PM conditions alone. $D_{\text{res}}$ isolated the portion of precipitation that is not predictable from the same covariates. As a diagnostic check on this parsing-out step, we regressed the residualized outcome $Y_{\text{res}}$ and residualized treatment $D_{\text{res}}$ on the covariate set *X*. We found near-zero R^2^ values for both residuals which confirmed minimal remaining dependence on *X*.

As a sensitivity check, we estimated a full-sample treatment effect to compare the overall removal rates between PM_2.5_ and PM_10_. Robust linear regression was used as an alternative to Ordinary Least Square (OLS) regression in the final stage as a few residuals can have an unusually large influence on model fitting. It provided a compromise between least-squares and absolute-error estimation (Huber 1964; Wang et al. 2022). The final-stage removal rate (θ) was determined using the following robust linear regression objective:

(7) $$\hat{\theta }=arg\underset{\theta }{min}\sum_{i=1}^{n}l\left(Y_{\text{res}},{\hat{Y}}_{\text{res}}\right)$$

where

(8) $${\overset{ˆ}{Y}}_{res}=\theta \cdot D_{res}$$

and

(9) $$l\left(Y_{res},\;{\hat{Y}}_{res}\right)=\left\{\begin{array}{cc} {\left(Y_{res}-{\hat{Y}}_{res}\right)}^{2}, & \left|Y_{res}-{\hat{Y}}_{res}\right|\leq \delta \\ 2\delta \left|Y_{res}-{\hat{Y}}_{res}\right|-\delta^{2}, & \left|Y_{res}-{\hat{Y}}_{res}\right|>\delta \end{array}\right.$$

The final-stage regression was fitted without an intercept term following the partialling-out logic used in Double Machine Learning (Chernozhukov et al. 2018). The θ parameter represents the estimated precipitation-removal rate, and δ is the HUber-loss (*l*) threshold selected using cross-validation for each precipitation-intensity, initial PM concentration and smoke intensity bin. For smaller residuals (≤ δ), *l* behaves like mean squared error (MSE) and for large residuals (> δ), *l* behaves like mean absolute error (MAE).

Precipitation and initial PM concentration were binned to different groups based on intensity and concentration, respectively. We created 0.5 mm equal interval bins for the light precipitation range (0 - 2.5 mm) as defined by AMS (2026) because of large variation in this range. Precipitation intensities above this range were binned at 1 mm intervals up to 15 mm and all precipitation bins exceeding 15 mm were assigned to a single upper bin. Initial PM concentration was binned similarly. Concentration values below 5 μg/m^3^ were binned into one low concentration bin and concentrations from 5 – 40 μg/m^3^ were divided into intervals of 2.5 μg/m^3^. Values above 40 μg/m^3^ were placed in a single upper bin. This helped to preserve details in lower and mid-range values and reduced the instability in higher value bins caused by low sample counts. To analyze PM removal across baseline PM concentration, observations were grouped by baseline PM concentration, and the APCP distribution in each PM bin was matched to that of a reference PM bin using nearest-neighbor matching. Another reason for this binning was to avoid data dilution in bins that might be introduced by other binning strategies like quantile binning (Zhao et al. 2020). We estimated removal rate (θ) and total removal (θ × APCP) for each bin. The 95% confidence intervals were constructed using the asymptotic standard errors from the fitted robust regression model as ± 1.96 standard errors.

### Comparison between removal estimates and wet scavenging theory

To compare the machine-learning-based removal estimates with existing wet-scavenging schemes (Choi et al. 2020; Jung et al. 2023; Wang et al. 2014), the binned expected PM removal was fitted using an exponential washout model. The previous wet-scavenging studies treat below-cloud removal as a first-order decay process, where pollutant concentration decreases exponentially with precipitation and the decay rate is represented by a scavenging coefficient (Λ). This coefficient is commonly parametrized as a power law:

(10) $$\Lambda \;(APCP)=a\cdot {APCP}^{b}$$

Assuming first-order decay of PM concentration during precipitation,

(11) $$PM_{t}=PM_{t-1}e^{-a\cdot {APCP}^{b}}$$

and

(12) $$\%{removal}_{\text{fit}}(APCP)=\left(e^{-a\cdot {APCP}^{b}}-1\right)\times 100$$

Parameters *a* and *b* were estimated separately for PM_2.5_ and PM_10_ by fitting the nonlinear washout model to the binned mean percent removal as a function of the median APCP within each precipitation-intensity bin.

## Results and Discussion

### Hourly PM concentrations

Average PM concentrations were compared between hours with and without precipitation using an APCP threshold of 0.1 mm. Nationwide average PM_2.5_ was 5.92 μg/m^3^ during precipitation hours, compared with 8.30 μg/m^3^ during non-precipitation hours. Similarly, average PM_10_ was 12.30 μg/m^3^ during precipitation hours, compared with 23.32 μg/m^3^ during non-precipitation hours. These summary statistics suggest lower concentrations during precipitation periods and suggest more efficient wet removal of PM^10^ compared to PM^2.5^.

We compared the PM concentration across all monitoring locations during the wildfire and non-wildfire period (**Error! Reference source not found.**). Median PM_2.5_ concentrations increased consistently from a baseline of 6.5 μg/m^3^ under non-smoke (NS) conditions to 16.9 *μ*/m^3^ during heavy smoke events (approximate 2.6-fold increase). The interquartile range (IQR) also widened substantially with smoke severity. The third quartile (Q3) for heavy smoke was 34.1 μg/m^3^ which is more than 3 times the Q3 value of 10.2 μg/m^3^ observed in the absence of wildfire smoke. A similar but larger increase in concentration with smoke density was seen for PM_10_, with a wide distribution in PM_10_ under heavy smoke conditions. Median PM_10_ concentration increased from 15.9 *μ*/m^3^ in the no-smoke group to 42 *μ*/m^3^ in the heavy smoke group, a 2.6-fold increase consistent with the increase in PM_2.5_. The change in PM concentration is consistent with earlier studies showing that wildfire smoke is a major source of particulate matter pollution (Aguilera et al. 2021a; Liu et al. 2017; Zhang et al. 2023a). Higher PM_10_ concentrations under heavy smoke show the mixed size distribution of wildfire plumes, which includes fine particles, coarse ash, dust, and other material generated during active burning and transport (Schweizer et al. 2019). A detailed percentage comparison of PM variation across all smoke levels is provided in the supplementary material (**Error! Reference source not found.** and **Error! Reference source not found.**).

### Spatial variation of unadjusted removal efficiency

Unadjusted median PM_2.5_ and PM_10_ removal efficiency in hours with at least 0.1 mm of precipitation is not spatially random (Figure 4). High removal efficiency clusters are concentrated across the eastern U.S., particularly across the Mid-Atlantic, Southeast, and Central US. The Eastern U.S. experienced more frequent precipitation (Karl and Knight 1998; Kunkel et al. 2010), higher cloud liquid water content (Harding and Snyder 2014), and a dominance of stratiform precipitation associated with midlatitude frontal systems (Liu et al. 2013; Song, Zhang, and Li 2012). In addition, PM_2.5_ in the eastern U.S. was historically high in secondary sulfate, which is highly soluble (Chan, Gantt, and McDow 2018), although the relative contribution of sulfate has declined in recent years as emissions controls and reduced coal use have led to lower SO_2_ emissions and increased relative importance of nitrate and organic aerosol (Hand et al. 2024).

The Local Μoran’s I clustering for PM_10_ is weaker and patchier than PM_2.5_ (Figure 4), with smaller groups of significant high-removal and low-removal areas. Larger particles that make up most of the PM_10_ mass are typically composed of crustal and road dust, whose emissions depend on local traffic, road conditions, wind, and surface state (Masri, Kang, and Koutrakis 2015). In the West and Southwestern US, the windblown desert dust and playa emissions are the major sources of PM_10_, and these events are intermittent in nature and less likely to co-occur with precipitation (Yin et al. 2005). Precipitation frequency is low across large portions of the interior West, and it has been decreasing in the past decade (Nash, Ye, and Fetzer 2017). California receives about 75% of its annual precipitation from November through March in a small number of large storms and atmospheric rivers (OEHHA 2025). These conditions reduce the number of precipitation events available to remove particles, which lowers cumulative PM removal, likely contributing to low-removal clusters in areas like Southern California for both PM_2.5_ and PM_10_.

### Estimates of PM changes associated with precipitation

Model evaluation showed stable residual distributions across the five folds for both PM_2.5_ and PM_10_, with similar medians and interquartile range. We did not observe any major differences in $\overset{ˆ}{g}(X),\hat{m}(X)$, or the main effect ($\hat{\theta }$) across folds (**Error! Reference source not found.** and **Error! Reference source not found.**). PM_10_ had consistently larger negative effects than PM_2.5_ across all cross-validation folds.

The results indicate that precipitation removes both PM_10_ and PM_2.5_, and the removal process is nonlinear across precipitation rates and different for PM_10_ and PM_2.5_ (Figure 5). The estimated removal rate $\left(\overset{ˆ}{\theta }\right)$ becomes more negative with increasing precipitation up to approximately 1–2 mm. At this range, the PM_10_ removal rate reaches −7% mm^−1^, and PM_2.5_ removal rate is smaller in magnitude −3% mm^−1^. Beyond this threshold, $\overset{ˆ}{\theta }$ reduces in magnitude at higher precipitation intensity, indicating that PM removal became less efficient per additional millimeter of precipitation at higher intensities.

Total removal increased in magnitude with increased precipitation intensity across all precipitation bins for both PM_2.5_ and PM_10_ (Figure 5). For example, under light precipitation (< 0.5 mm), total estimated PM_10_ removal was −1.98 (−1.10, −1.71) % and was −0.22 (−0.34, 0.02) % for PM_2.5_. At a moderate precipitation range of 3-4 mm, total estimated PM_10_ removal decreased to −16.98 (−14.62, −15.80) % and PM_2.5_ removal decreased to −10.13 (−9.34, −10.91) %. At higher intensity rainfall (around 15 mm), total estimated PM_10_ removal decreased further to −24.58 (−22.64, −24.58) % and PM_2.5_ removal decreased to −17.79 (−16.38, −19.19) %. Although removal rate declined in magnitude after approximately 2.5 mm hr-^1^, total removal increased in magnitude with more rainfall.

Overall average removal rate was more negative for PM_10_ (−2.10 (−2.16, −2.03) %) than PM_2.5_ (−1.41% (−1.45, −1.37) %) and removal rates were stronger for PM_10_ than PM_2.5_ across all precipitation bins. This difference is consistent with established wet scavenging theory. Coarse particles have larger aerodynamic diameters and greater effective cross-sectional area. This increases collision efficiency through inertial impaction and interception. Fine particles remain more tightly coupled to the surrounding airflow and are more likely to be removedthrough diffusional processes driven by Brownian motion (Figure 1). The diffusion process is less efficient under light to moderate rainfall conditions (Seinfeld 2016; Slinn 1983). Size-resolved modeling work by Petroff and Zhang (2010) and Wang et al. (2010) also reported that scavenging efficiencies for coarse particles can exceed those of fine particles because of the stronger inertial impaction and interception.

Unadjusted removal rates show that precipitation combined with other meteorological factors has a limited effect on PM when the particle mass concentration is below 5 *μ*/m^3^ for PM_2.5_ and 7.5 *μ*/m^3^ for PM_10_ (Figure 6, left). However, the isolated effects of precipitation on PM concentrations are negative even at low concentrations and increase with increasing concentrations. (Figure 6, right). Rainfall removal of PM_10_ is again more efficient than PM_2.5_ across concentrations. For example, the modeled removal rate of PM_2.5_ at initial concentration of <5 *μ*/m^3^ was −1.03 (−0.92, −1.14) % and at >40 *μ*/m^3^ was −6.76 (−5.72, −7.80) %. The removal rate of PM_10_ at the same initial concentrations were −2.43 (−2.14, −2.71) % and −14.50 (−13.93, −15.07) %, respectively. We did not observe a saturation point or corresponding concentration threshold, and the removal continues to increase with PM concentrations within the observed data ranges.

These findings indicate that the relationship between precipitation and PM removal depends on both the intensity of precipitation and the initial PM concentration, with greater removal efficiencies in PM_10_ than PM_2.5_. This aligns with the results obtained in a study of a single storm event by Chate et al. (2003) and over a 1-year period in Chengdu, China by Li et al. (2025). The influence of particles on the collision and attachment rates between falling raindrops is dependent on particle availability because the probability of a raindrop intercepting a particle increases at higher baseline concentration. This phenomenon shows that precipitation removal efficiency increases with increasing PM concentrations, as discussed in previous literature(Chandrakar et al. 2024; X. Zhao et al. 2020).

### Removal effect under the influence of wildfire smoke

To compare the distribution of PM precipitation removal rate across four categories of wildfire smoke, we matched distributions of initial PM concentration before estimating the removal rate. Heavy smoke had the smallest sample size and was used as the reference group. For other smoke categories, nearest-neighbor matching was applied to select observations with initial PM concentrations close to those observed under heavy smoke. The matched distributions of baseline PM concentrations are similar across all smoke groups for each particle size category, and each smoke category contained the same number of observations as the heavy-smoke group (**Error! Reference source not found.**).

The removal rates of both PM_2.5_ and PM_10_ are larger in magnitude under no-smoke conditions and become less negative as smoke density increases (Figure 7). Removal rates were more negative for PM_10_ than for PM_2.5_, consistent with the size dependence identified in the main analysis. In sensitivity analyses assessing removal under smoke conditions for precipitation ranges of 1-3 mm, 2-4 mm, and 3-5 mm, results are consistent (**Error! Reference source not found.**).

More efficient PM removal by precipitation under low smoke conditions can be attributed to smoke particles acting as Cloud Condensation Nuclei (CCN), which reduce cloud droplet size, which in turn suppresses the collision-coalescence process, as supported by references et al. 2009; Lu and Sokolik 2013). The chemical and physical properties of particles present in smoke conditions likely further influence wet removal. Biomass burning smoke particles are mostly made of organic material (with 5–20% elemental carbon and at least 50% organic carbon (Aguilera et al. 2021b)), usually internally mixed with some inorganic species (Gomez et al. 2018). Although some of them are water-soluble, most of the smoke particles usually have a low but non-negligible hygroscopicity compared to other aerosol compounds like sulfate and nitrate (Petters et al. (2009) and (Gregory et al. 2007). This phenomenon leads to weaker precipitation scavenging under high-smoke conditions under similar distributions of initial PM concentrations and precipitation.

### Comparison with existing scavenging schemes

We compared the results of the DoubleML model with previous wet-scavenging studies by fitting estimated total removal as a power law in Equation (12) across precipitation bins (Figure 8). The fitted equation was used to derive the empirical scavenging parameters *a* and *b*. The estimated coefficients were *a* = 0.0404 and *b* = 0.5951 for PM_2.5_ (R^2^= 0.93) and *a* = 0.0812 and *b* = 0.4508 for PM_10_ (R^2^= 0.92).

The fitted coefficients *a* and *b* varied across previous studies depending on factors such as study precipitation duration, particle size distribution, and the physical assumptions used in each analysis (Table 1). Our estimated washout coefficients fall within the range reported in prior studies. A likely source of discrepancies between this study and previous studies is the difference in PM size categories used and methodology to derive the estimates. Most existing studies derive washout coefficients from theoretical scavenging models or controlled laboratory experiments and rely on idealized assumptions about droplet size distributions and collection efficiency. Some of the comparison studies like Wang, Zhang, and Moran (2014) and Feng (2007), themselves vary by an order of magnitude. Our coefficients are estimated directly from a decade of observational AQS and NLDAS data using a data-driven causal inference framework. As such, our estimates are not constrained by the simplifying assumptions of theoretical or experimental parameterizations and may better reflect real-world removal behavior.

Estimated total removal follows the power law for precipitation magnitudes larger than about 2 mm hr^−1^, but for precipitation below 2 mm hr^−1^, removal rates are well below what is expected by traditional theory. In a sensitivity analysis using APCP > 2 mm, values for a and b were similar and slightly larger in this sensitivity analysis compared to the main analysis. The fit was stronger than in the primary analysis with R^2^ = 0.94 for PM_2.5_ and R^2^ = 0.96 for PM_10_ (Table 1 and **Error! Reference source not found.**), showing that removing observations of low precipitation amounts better agrees with proposed theory. This may be due to uneven precipitation within NLDAS grid cells, which may be more magnified at low precipitation rates. NLDAS Precipitation is reported at hourly and 1/8° grid resolution, so a value below 2 mm hr^−1^ may represent light, spatially averaged precipitation across a grid cell rather than rainfall at the monitor location (Cosgrove et al. 2003). In addition, precipitation droplet size is likely to be smaller at lower precipitation rates, leading to fewer interceptions and impactions with particles.

### Limitations

Although this work provides an estimate of the removal effect due to precipitation, several other factors can still vary like the chemical composition of aerosol and the droplet size distribution, which were not incorporated in our analysis. These properties might affect the droplet activation and solubility of aerosol during wet deposition (Jones et al. 2022; Niu et al. 2023). Similarly, the HMS smoke dataset used in this study is an operational dataset based on satellite imagery which can introduce spatial uncertainty and the smoke intensity category might not fully capture aerosol loading intensity or vertical distribution (Brey et al. 2018b). This is a limitation because HMS identifies daytime column smoke rather than smoke at the surface, so it may not match ground-level PM exposure as measured in EPA monitors, but previous studies and our assessment (**Error! Reference source not found.**, **Error! Reference source not found.** and **Error! Reference source not found.**) show that HMS smoke categories correlated with increased PM concentrations. In addition, this analysis used total ambient PM concentrations rather than source-specific PM, even though the removal rates might alter across source types because emission sources such as traffic, biomass burning, dust, and secondary aerosols produce particles of different chemical and physical properties (Dadashazar, Ma, and Sorooshian 2019). A rainy hour may still show only a small drop in PM from (t−1) to *t* because rainfall intensity can vary sharply within the hour.

The Double ML approach used in this study assumes that relevant confounders are observed and that precipitation has sufficient overlap with PM concentrations and meteorological conditions. Confounding that is not observed in the data, sparse data at high precipitation or heavy smoke, and sensitivity to nuisance model specification may affect the estimates, so the results should be interpreted within these assumptions. Hence, the estimated removal effect should be interpreted as a bulk response, with the potential for overestimation when smoke conditions are dominated by fine-particles (PM_2.5_) and underestimation when coarse particles (PM_10_) contribute more to ambient PM.

## Conclusions

This study provides a data-driven assessment of particulate matter wet scavenging across the contiguous United States. Ultimately, the empirical power-law washout coefficients derived herein (a = 0.0404, b = 0.5951) for PM_2.5_ and (a=0.0812, b=0.4508) for PM_10_ provide observation-based parameters for chemical transport models. Our work advances the understanding of PM wet scavenging in several ways. First, we used ten years of hourly observations from 760 PM_2.5_ and 451 PM_10_ monitoring stations to derive precipitation removal, an advancement over previous studies at a small number of locations or in laboratories. Second, the DoubleML method applied in this study adjusts for non-linear relationships and confounding with other meteorological variables. In addition, our analysis quantified how the influence of precipitation on wet scavenging changes under wildfire smoke and non-wildfire PM conditions. These parameters overcome the limitations of localized or theoretical models and are applicable to assessment studies of human exposure to PM, air quality model calibration and PM deposition to land, vegetation, and surface waters.

Each additional millimeter of precipitation above 2.5 mm removed PM less efficiently even though total PM removal continued to increase. This effect also depended on the size of the aerosol as PM_10_ was removed more efficiently than PM_2.5_ under similar initial concentration and precipitation. We did not observe a saturation point within the studied concentration range. Our results show that aerosol mass removal by precipitation increases with intensity of precipitation, particle size, and initial concentration of particles. Wildfire smoke loading reduces the efficiency of PM removal compared with no-smoke conditions at similar PM levels and precipitation conditions. We observed the largest removal rate at precipitation intensities of 1 to 2 mm although the total PM removal remained small because of the low precipitation amount.

## Supplementary Material

This is a list of supplementary files associated with this preprint. Click to download.

- SupplementaryInformation.docx
- SupplementaryInformation20260830.docx

## Figures and Tables

**Figure 1:**
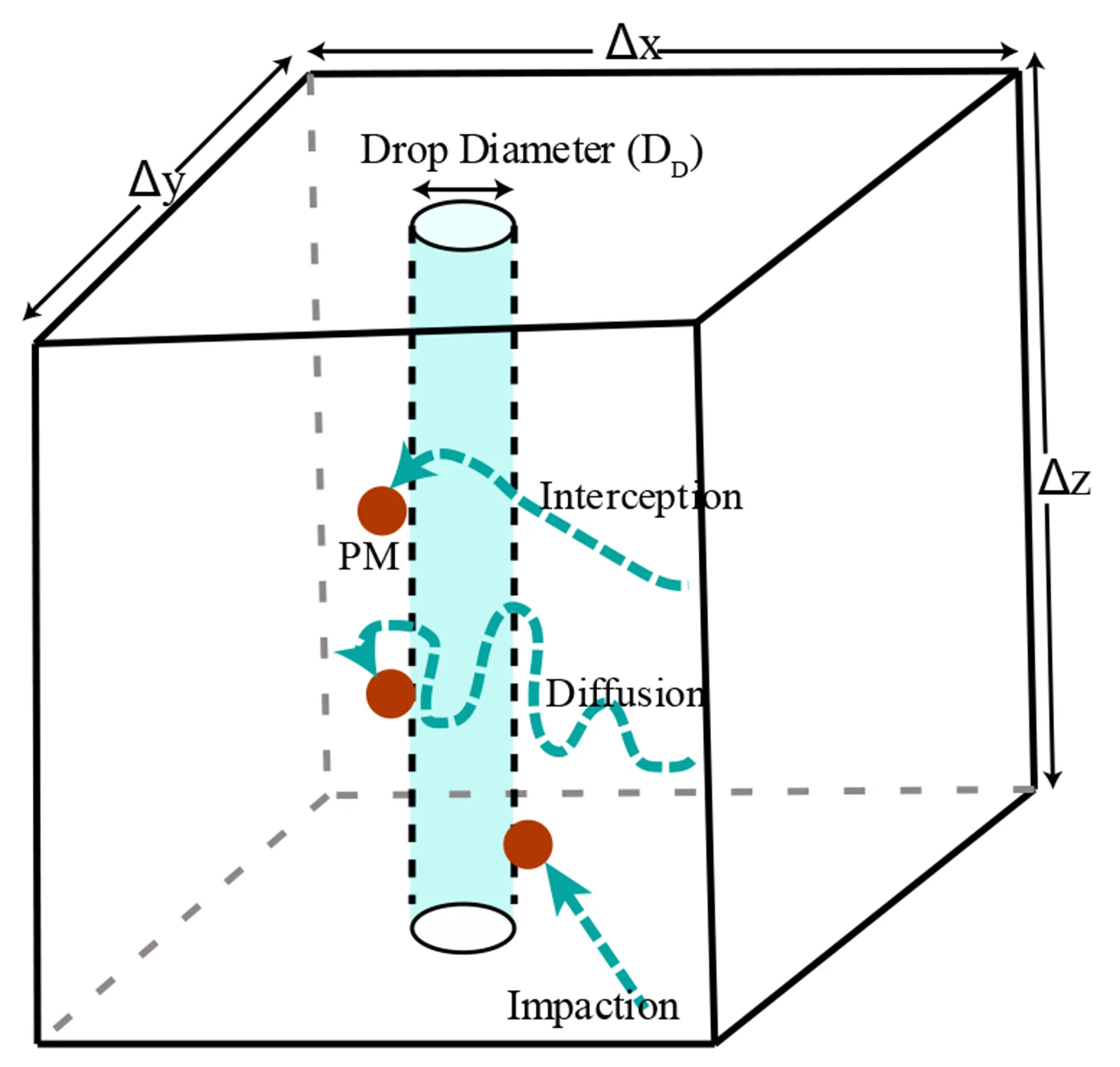
PM removal by precipitation, where raindrops with diameter (D_D_) capture airborne particles within a control volume (Δx, Δy, Δz) through impaction, interception, and diffusion.

**Figure 2:**
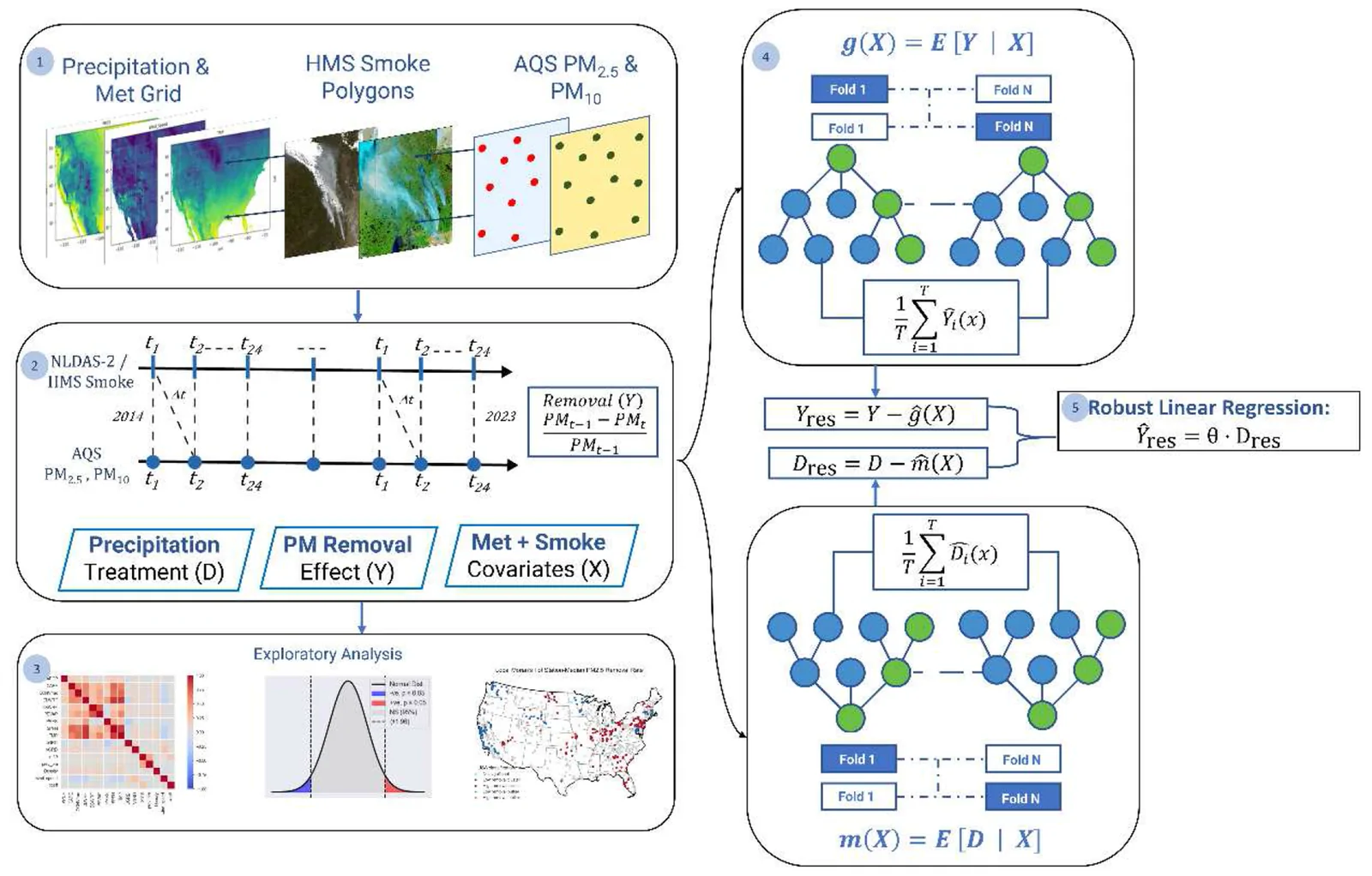
Methodological framework for estimating the conditional effect of precipitation on PM removal. (1) Hourly AQS PM observations were linked with NLDAS-2 meteorology and HMS smoke polygons. (2) These data defined precipitation treatment D, PM removal outcome Y, and meteorological and smoke covariates X. (3) Exploratory analyses were used for variable relationships and spatial patterns. (4) Cross-fitted nuisance models estimated residuals of outcome and treatment. (5) These residuals were used in a final-stage linear regression to estimate $\hat{\theta }$

**Figure 3:**
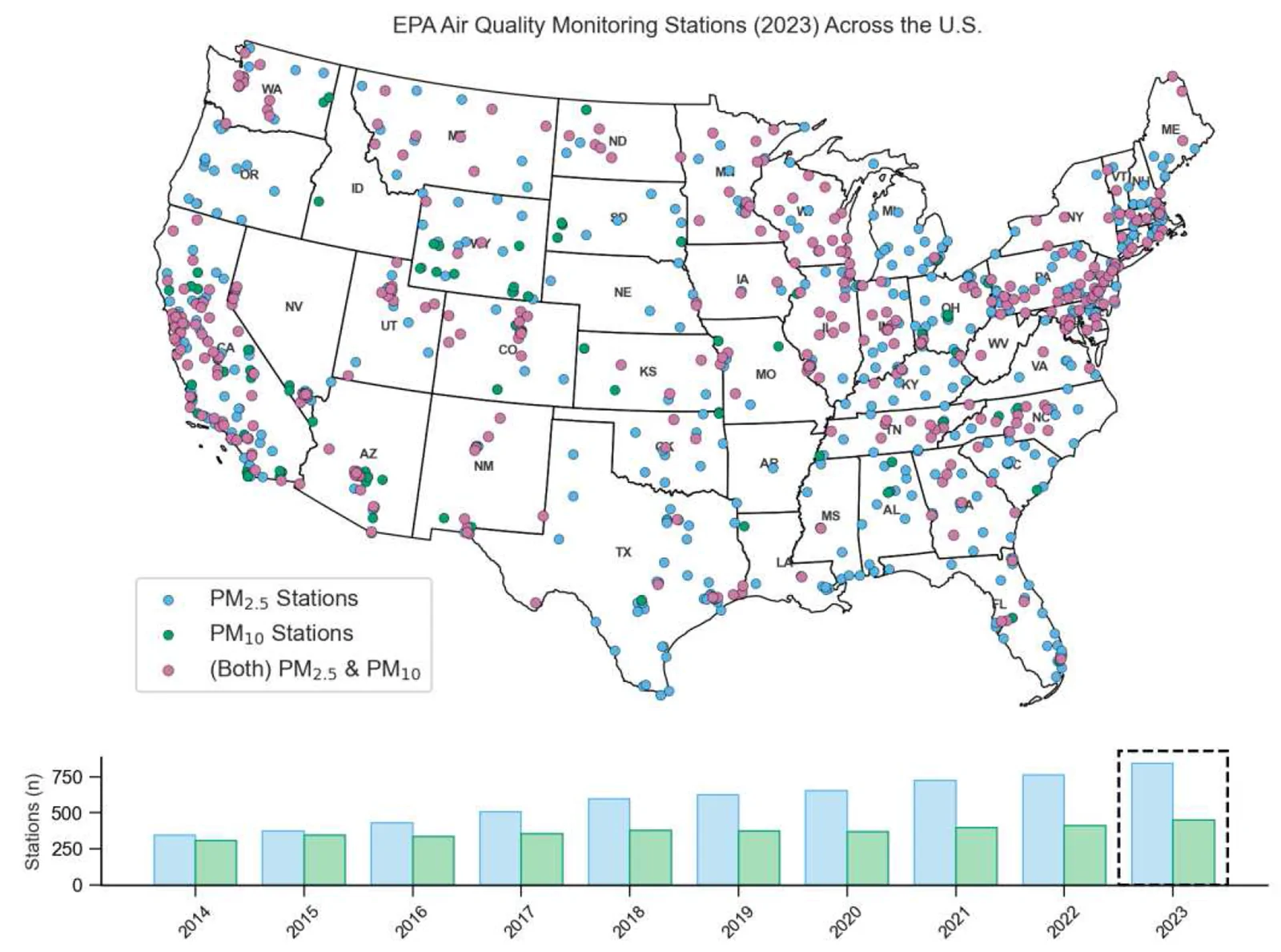
The spatial distribution of EPA monitoring stations across the contiguous United States in 2023 (top) and the number of PM_2.5_ and PM_10_ monitoring stations in each year from 2014 to 2023 (bottom).Station locations are classified as PM_2.5_ only, PM_10_ only, or co-located PM_2.5_ and PM_10_ monitors.

**Figure 4:**
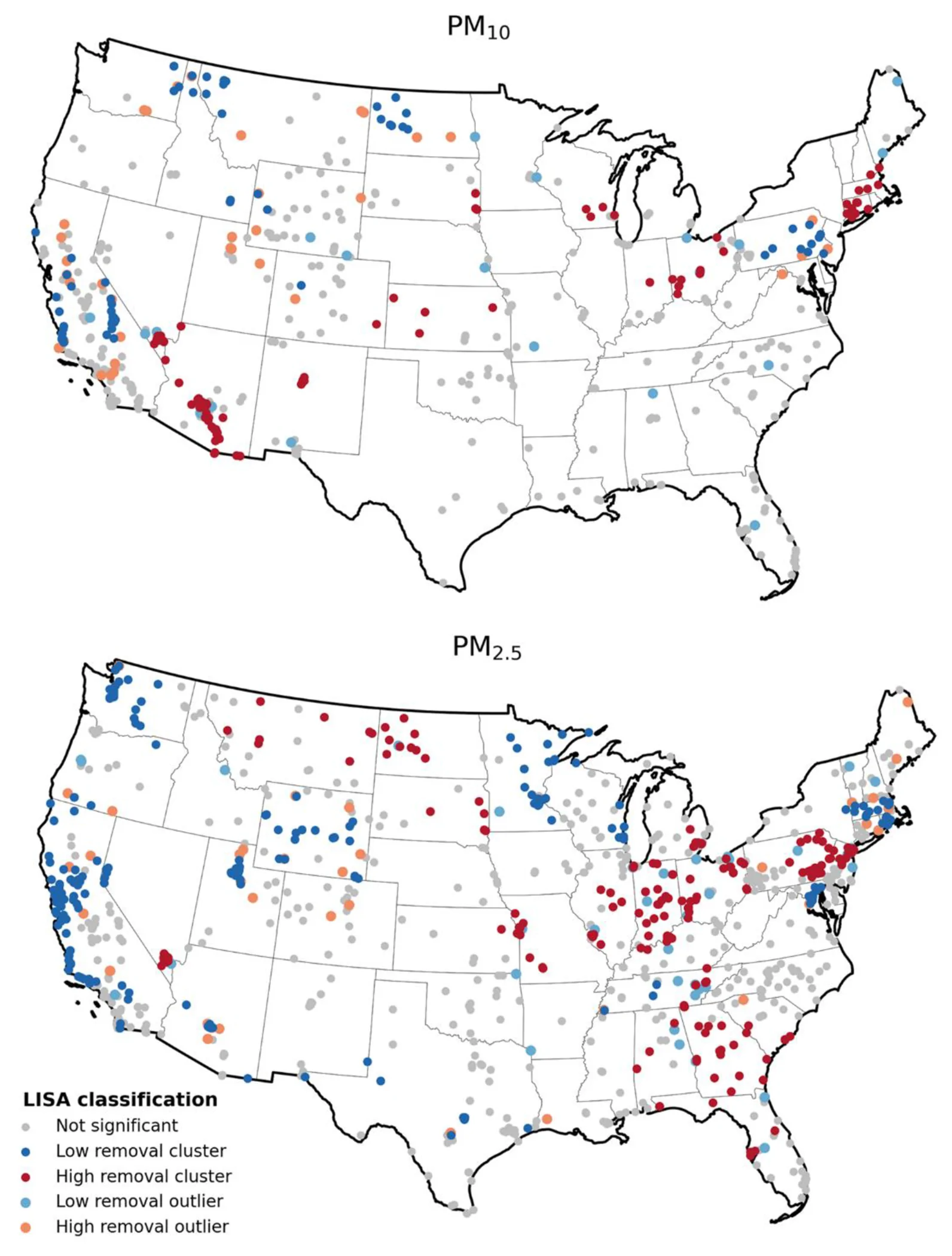
Local Moran’s I statistics for station-median precipitation-driven removal rates of PM_2.5_ (bottom) and PM_10_ (top) across the contiguous U.S. Stations are classified as high-removal clusters, low-removal clusters, high-removal outliers, low-removal outliers, or not significant based on permutation-based significance testing (p < 0.05). A cluster represents neighboring stations with similar removal pattern as described in Eq. (2).

**Figure 5:**
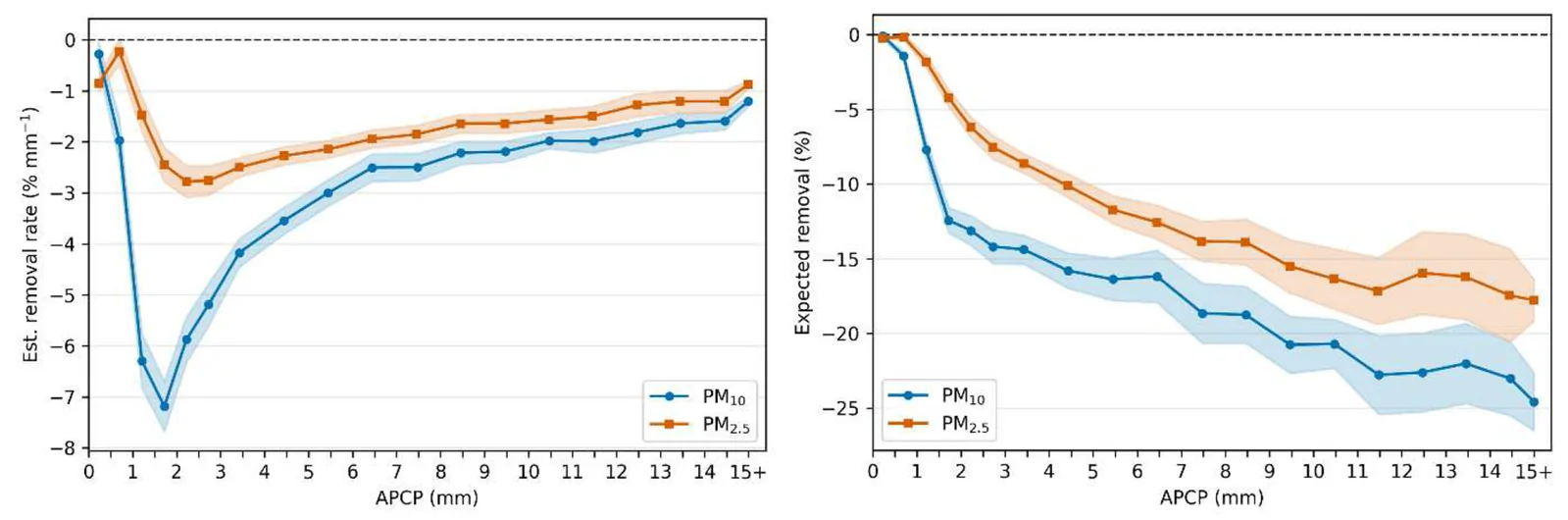
Estimated average removal rate of precipitation ($\hat{\theta }$) on PM_2.5_ and PM_10_ across precipitation intensity (APCP) (right) and total removal in percentage (left). Shaded bands indicate 95% confidence intervals within each bin.

**Figure 6:**
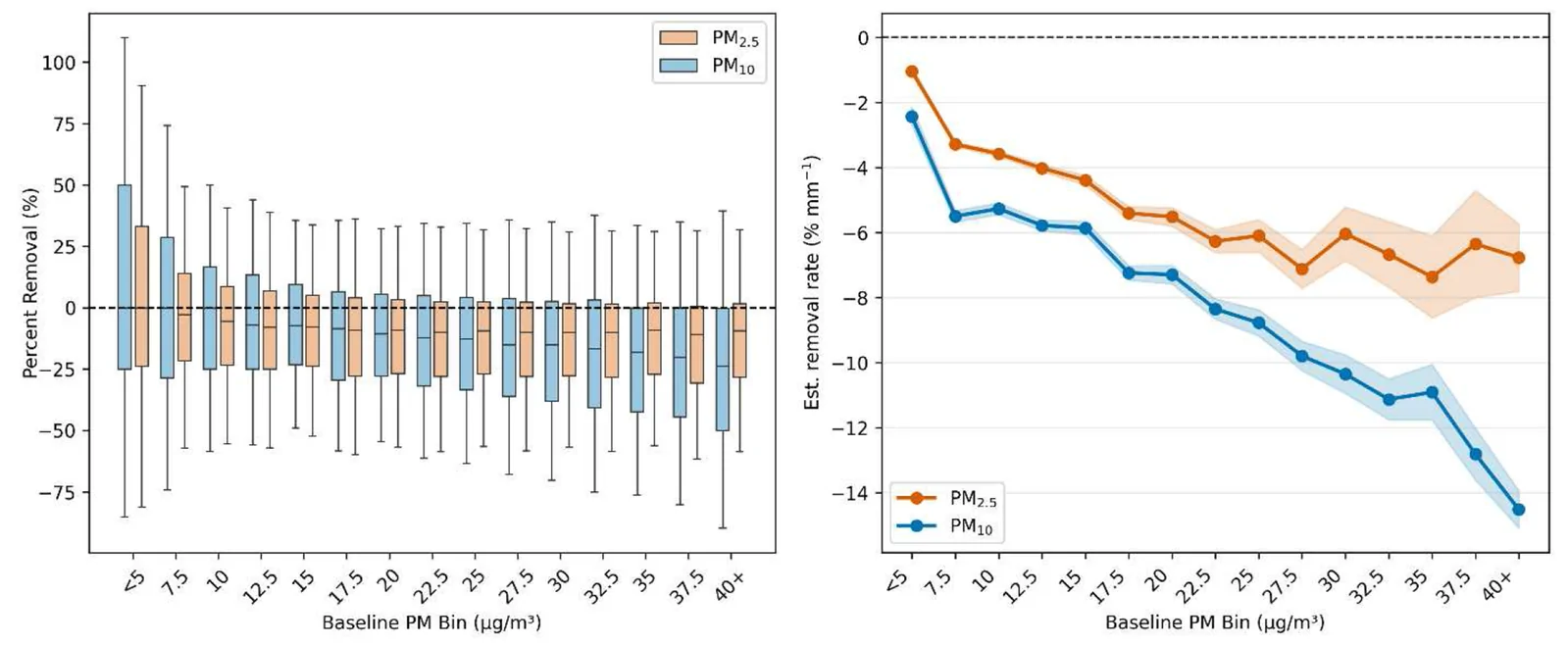
Overall percentage removal by baseline PM concentration (left) and modeled removal rate across different initial PM concentrations (right) for PM_2.5_ and PM_10_.

**Figure 7:**
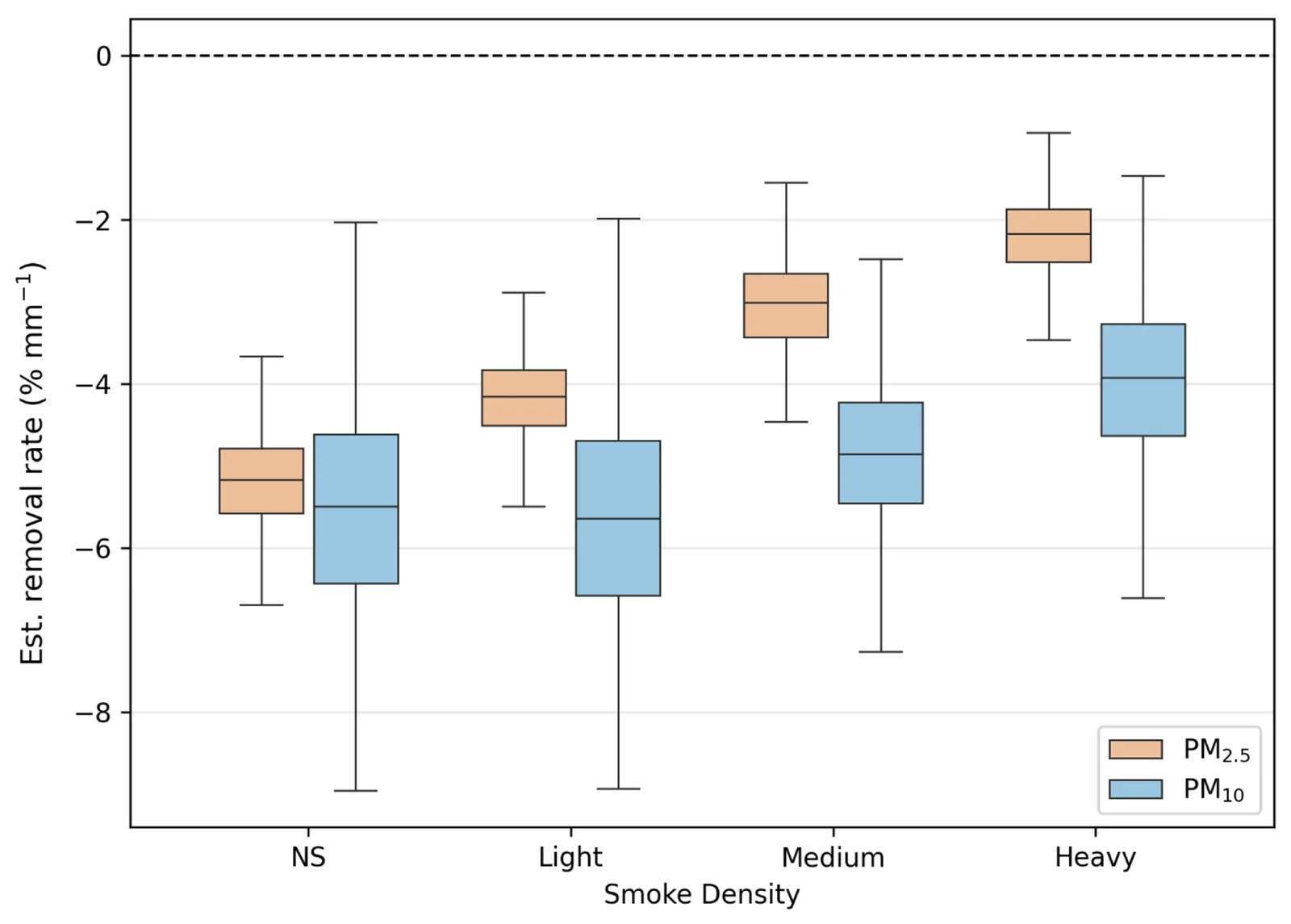
Estimated PM_2.5_ (N=3492) and PM_10_ (N=1552) removal rates across wildfire smoke density categories under full APCP distribution, with PM concentrations matched to values in the Heavy smoke category. Negative values indicate stronger removal.

**Figure 8:**
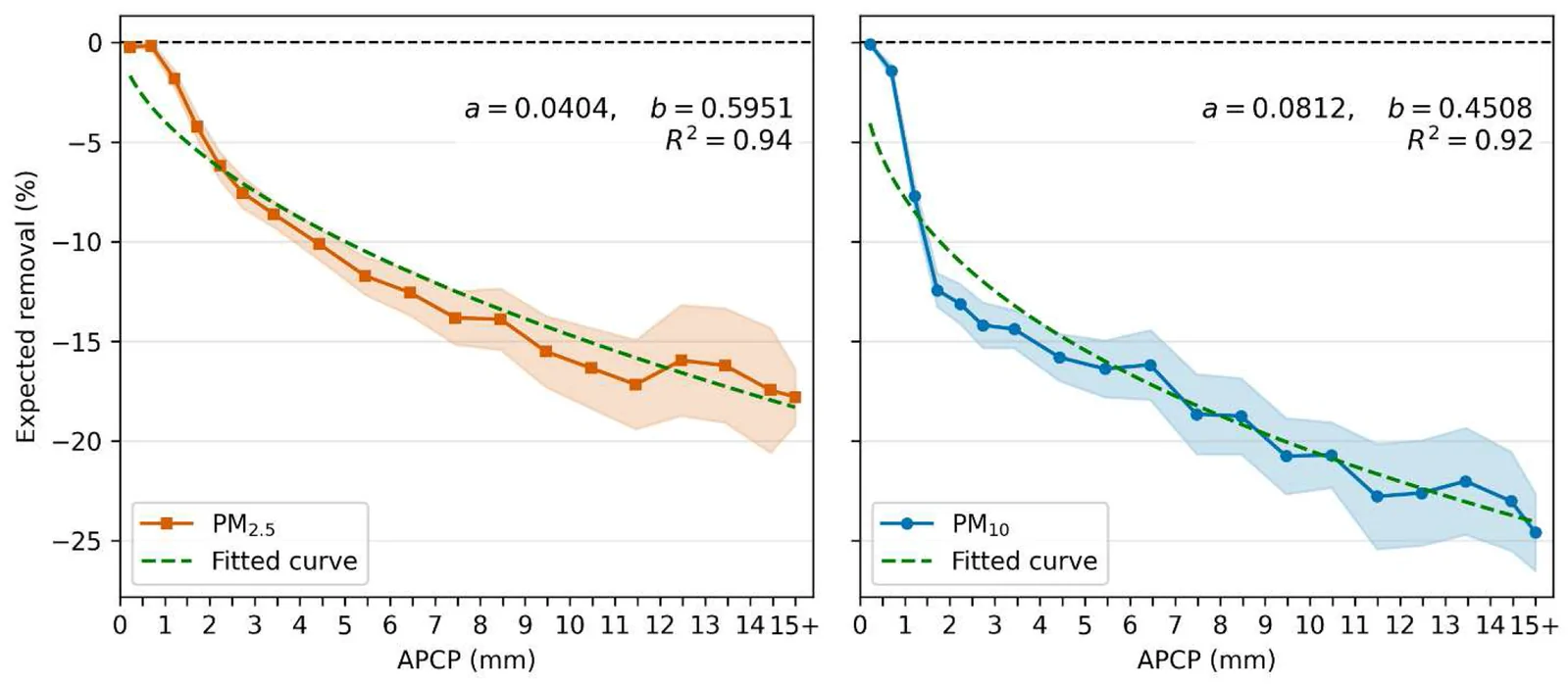
Expected removal of PM_2.5_ (left) and PM_10_ (right) as a function of precipitation intensity APCP. Shaded bands show 95% CI. Dashed curves show the fitted power-law washout model with fitted a and b reported in each panel from Equation (12).

**Table 1:** washout coefficients (a and b) of comparable studies, with APCP expressed as mm hr^−1^. GSD refers to geometric standard deviation

Reference	a (hr^−1^)	b	Note
This study	0.0404	0.5951	PM_2.5_ (R^2^ = 0.93)
	0.0812	0.4508	PM_10_ (R^2^ = 0.92)
This study (APCP > 2 mm)	0.0531	0.4819	PM_2.5_ (R^2^ = 0.94), Sensitivity Analysis
	0.1016	0.3536	PM_10_ (R^2^ = 0.96), Sensitivity Analysis
(Choi et al. 2020)	0.07308	0.543	Bulk PM
(Jung et al. 2023)	0.0144	0.9268	Observation based, PM_10_
	0.0632	0.3353	Analytical, mean diameter 2.5 μm, GSD of 2.3 μm
	0.1959	0.3261	Analytical, mean diameter 2.5μm, GSD of 2.5 μm
(Andronache 2003)	0.24	0.7	Bulk PM
(Wang et al. 2014)	0.0014 – 0.0221	0.67 – 0.72	Semi-empirical, size: 0.01 – 2 μm
	0.243 – 2.48	0.82 – 0.91	Semi-empirical, size: 3 – 100 μm
GEOS-CHEM	0.00106	0.61	Fine Mode
	1.57	0.79	Coarse Mode
(Feng 2007)	0.0008 – 0.0013	0.61 – 0.62	Size: 0.04 - 2.5 μm
	0.7596 – 1.23	0.71	Size: 2.5 - 16 μm

## Data Availability

EPA AQS Data are publicly available at https://aqs.epa.gov/aqsweb/airdata/download_files.html HMS Smoke Data is available at https://www.ospo.noaa.gov/products/land/hms.html#data NLDAS-2 data is publicly available at https://ldas.gsfc.nasa.gov/nldas/nldas-2-model-data

## Code availability

The code supporting this study will be made publicly available through a GitHub repository (https://github.com/DineshNeupane/PptPMInteraction ) upon publication. For peer review, the complete code is included as a ZIP file with the submission.
